# Phylogenetic analysis and pathogenicity of H3 subtype avian influenza viruses isolated from live poultry markets in China

**DOI:** 10.1038/srep27360

**Published:** 2016-06-07

**Authors:** Hongrui Cui, Ying Shi, Tao Ruan, Xuesong Li, Qiaoyang Teng, Hongjun Chen, Jianmei Yang, Qinfang Liu, Zejun Li

**Affiliations:** 1 Shanghai Veterinary Research Institute, Chinese Academy of Agricultural Sciences, Shanghai, People’s Republic of China

## Abstract

H3 subtype influenza A virus is one of the main subtypes that threats both public and animal health. However, the evolution and pathogenicity of H3 avian influenza virus (AIV) circulating in domestic birds in China remain largely unclear. In this study, seven H3 AIVs (four H3N2 and three H3N8) were isolated from poultry in live poultry market (LPM) in China. Phylogenetic analyses of full genomes showed that all viruses were clustered into Eurasian lineage, except N8 genes of two H3N8 isolates fell into North American lineage. Intriguingly, the N8 gene of one H3N8 and PB2, PB1, NP and NS of two H3N2 isolates have close relationship with those of the highly pathogenic H5N8 viruses circulating in Korea and United States, suggesting that the H3-like AIV may contribute internal genes to the highly pathogenic H5N8 viruses. Phylogenetic tree of HA gene and antigenic cross-reactivity results indicated that two antigenically different H3 viruses are circulating in LPM in China. Most of the H3 viruses replicated in mice lung and nasal turbinate without prior adaptation, and the representative H3 viruses infected chickens without causing clinical signs. The reassortment of H3 subtype influenza viruses warrants continuous surveillance in LPM in China.

Influenza A virus (IAV), a member of the genus *Orthomyxovirus*, is classified into subtypes according to the serological reactivity of their surface glycoproteins, hemagglutinin (HA) and neuraminidase (NA)^1^. During the last 100 years, three subtypes of IAVs adapted to human had resulted in four appalling pandemics: H1N1 in 1918 and 2009, H2N2 in 1957 and H3N2 in 1968^2^,^3^,^4^. Moreover, H5, H7, and H9 subtypes had resulted in more than one thousand human infections over the last decade, which dramatically changed the perception and impact of avian influenza viruses in mammals^5^. For a long period, wild aquatic birds have been considered as the only natural reservoir for influenza A viruses. However, the recent description of H17N10 and H18N11 in new world bats has suggested other reservoir species may also exist^6^,^7^. With the constant emergence of novel reassortant influenza viruses and their interspecies transmission, IAV has become an important virus of great concerns and causes disease in humans, pigs, horses, and fowl^8^,^9^.

H3 subtype influenza viruses, known as having wide host range from birds to various mammalian species including humans, pigs, dogs, and horses^10^, are easier to evolve as a result of antigenic drift and antigenic shift^11^. Recent studies reported that some novel H3 subtype viruses reassorted with H5N1 and H9N2 in Korea and China^12^,^13^,^14^. Daesub Song reported cross species transmission of an intact avian influenza virus (H3N2) to dogs and caused acute respiratory disease in dogs in South Korea^15^. In addition, phylogenetic and epidemiologic analyses indicated that avian and human H3 subtype influenza viruses have transmitted to pigs and reassorted in pigs^16^,^17^. Moreover, several studies in Korea and Southern China had determined that the avian-origin canine H3N2 viruses can spread among dogs through contact with increased pathogenicity and broad host ranges^15^,^18^,^19^. H3N8 virus also has spread from avian to mammals^20^. The first equine H3N8 infection cases were reported in 1963 in Miami^21^. Since then, horses infected with avian H3N8 virus have been reported in China, and dogs infected with equine H3N8 virus have been observed in Florida^22^,^23^. Recently an H3N8 virus infected seals and resulted in 162 deaths^24^. Aquatic birds are the natural reservoir of influenza A virus. Recently, a novel H3N2 avian influenza virus was isolated in domestic duck in China. Sequence analysis showed that this novel H3N2 contains genes from H7N3, and H7N7 viruses that had close relationship with H7N9 virus, which suggested that reassortment occurred between H3N2 with other subtype influenza viruses^25^. Therefore, H3 subtype influenza viruses pose a threat to both animal and human health.

In China, live poultry markets are also served as slaughterhouses in which chickens, ducks, quails, guineafowl, and pigeons etc. are kept and mixed for days. Comingling of different poultry species makes live poultry market a major source of AIV dissemination, reassortment and interspecies transmission^26^. Most of H7N9 human infection cases are associated with exposure to LPM, which was identified as the source of human H7N9 infection cases^27^,^28^,^29^. Multiple subtype influenza viruses in LPM enhance the reassortment possibility. The evolution and reassortant situation of H3 subtype influenza virus in LPM remain unclear. In the present study, seven H3 subtype AIV strains (four H3N2 and three H3N8) were isolated from domestic ducks and chickens in LPM in China. To better understand the evolution of these H3 influenza viruses, the full genomes of the H3 AIVs were sequenced and analyzed. Phylogenic analyses of all eight segments were conducted and the pathogenicity of these isolates in mice and chickens were evaluated.

## Materials and Methods

### Virus isolation

Oropharyngeal swabs were collected from fowls at live poultry markets in Shanghai, Jiangsu, Guangzhou, Zhejiang, Shandong and Hebei provinces during 2009 to 2011. Virus isolation was performed by inoculating of 9-day-old specific-pathogen-free (SPF) embryonated chicken eggs with filtered swab supernatant. Embryos died within 24 h after inoculation were discarded. The allantoic fluid was collected from embryonated chicken eggs 72 hours after inoculation, of which hemagglutination assays (HA) were run with 0.5% packed chicken red blood cells. Hemagglutination inhibition (HI) assays were used to determine the HA subtype by using a panel of positive serum against H1 to H16 subtypes. HI tests were performed in accordance to WHO guidelines. All HI assays were performed in duplicates. For further verification, subtype specific real-time RT-PCR was performed with HA-specific primers targeting H3 genes as previously reported^30^.

### Cells and growth kinetics

Madin-Darby Canine Kidney Cells (MDCK), Human lung adenocarcinoma epithelial cells (A549) and DF-1 cells were maintained in Dulbecco’s Modified Eagle Medium (DMEM) with 5% bovine calf serum (BCS), 1× L-glutamine and 1× antibiotics. Cells were inoculated with the respective viruses in MEM infecting medium containing 0.3% BSA (Sigma), 1 mg/mL TPCK-treated trypsin (Sigma) and 1× antibiotics. To analyze virus growth kinetics, confluent MDCK, A549 and DF-1 cells were infected with each virus at a multiplicity of infection (MOI) of 0.01. The supernatants from infected cells were collected at different time points (12, 24, 36 and 48 hours post infection) and viral titers were determined in embryonated chicken eggs.

### RT-PCR and gene sequencing

A QIAGEN Viral RNA Isolation Kit was used to extract vRNA from 140 μL allantoic fluid from infected SPF embryonated eggs. The isolated RNA was eluted into 30 μL diethylpyrocarbonate-treated water. Two-step RT-PCR was employed to amplify each viral gene segment. The first-strand cDNA was transcripted by using reverse transcriptase M-MLV (TaKaRa, Dalian, China) with universal primer (5′-AGCAAAAGCAGG-3′) for influenza A virus in a final volume of 20 μL per manufacturer’s protocol. Eight fragments (PB2, PB1, PA, HA, NP, NA, M and NS) of each virus were amplified respectively by using the Hoffman universal primers for influenza A virus as described previously^31^. Amplicons of the appropriate sizes were subsequently gel purified by using a DNA Gel Extraction Kit (Axygen, Hangzhou, China). The purified PCR products were sequenced by the GENEWIZ Biotechnology Company using the Sanger sequencing methodology. Sequences were assembled by using SEQMAN program (DNASTAR, Madison, WI, USA).

### Phylogenetic analysis and molecular characteristics

Multiple sequence alignment and the phylogenetic and evolutionary analyses were conducted by using MEGA6.0. Briefly, phylogenetic trees of all eight full length gene segments (PB2, PB1, PA, HA, NP, NA, M and NS) of seven H3 isolates were generated by applying the neighbor-joining method with Kimura’s two-parameter distance model and 1000 bootstrap replicates^32^. The trees of HA and NA genes included sequences of representative H3 subtype viruses from Eurasian and North American lineage available in the GenBank database; trees of other six full length segments (PB2, PB1, PA, NP, M and NS) consisted of major epidemic strains of H9, H7, H5, H10, and other subtype influenza viruses from Eurasian and North American lineage in GenBank database.

### HI assay and antisera generation

HI assays were used to determine the antigenic cross-reactivity among H3 viruses from different groups in phylogenetic trees of HA. Four representative isolates (74-1/H3N2, 120-1/H3N8, 854-2/H3N8 and B1646-2/H3N2) from group 1 and group 2 were chosen for antigenic cross-reactivity tests based on the phylogenetic trees of HA gene. Antisera against 74-1/H3N2, 120-1/H3N8, 854-2/H3N8 or B1646-2/H3N2 respectively were generated by immunizing SPF chickens twice with UV inactivated viruses. HI tests were performed in accordance to WHO guidelines.

### Pathogenicity in mice

Seven H3 subtype influenza viruses were purified and propagated by inoculating 9-day-old SPF chicken embryonated eggs. Allantoic fluid was harvested and titrated in 10-day-old SPF embryonated eggs to determine the 50% embryo infectious dose (EID_50_) by the Reed and Muench method^33^,^34^. To determine the pathogenicity of the H3 isolates, ninety-six 4 to 6-week-old female BALB/c mice (Vital River Laboratories, Beijing, China) were separated randomly in 8 groups (12 mice per group). Mice in each group were anesthetized and intranasally (i.n.) inoculated with 10^6^ EID_50_ of each virus in 50 μL phosphate-buffered saline (PBS). The mock group was inoculated intranasally with 50 μL of PBS. Body weights, clinical signs were monitored daily, and three mice from each group were euthanatized at 3 and 5 days post infection (dpi) respectively. Lung, spleen, nasal turbinate, heart, and brain tissue were collected aseptically. Then, the tissues were homogenized and the homogenates were titrated in 10-day-old embryonated eggs. For histopathological examination, tissue samples of lungs were fixed in 10% buffered formalin, and processed and stained with hematoxylin and eosin (H&E). The lung sections were scored by a pathologist in a blinded fashion. The scores assigned from 0 to 3 reflecting the severity of microscopic lung lesions were based on damage of epithelial layer, damage of alveolar septal and alveolar, and lymphocyte infiltration. Those statistical variables were subjected to comparisons for all pairs by using the Tukey–Kramer test. Pair-wise mean comparisons between inoculated and control groups were made using the Student’s t-test (GraphPad software Inc, CA); a P-value < 0.05 was considered statistically significant.

### Pathogenicity in chickens

To assess the pathogenicity of the isolated H3 AIVs in chickens, three representative H3 viruses (120-1/H3N8, B854-2/H3N8 and B1646-2/H3N2) from group 1 and group 2 in phylogenetic trees of HA were chosen for chicken study. Groups of seven 12-week-old SPF chickens were used, inoculated intranasally with 10^6^ EID_50_ in 0.1 mL PBS. All chickens were observed daily for clinical signs until 14 dpi. Oropharyngeal and cloacal swabs were collected at 3, 5, and 7 dpi from the infected chickens to detect viral shedding. To elucidate virus replication and distribution in the infected chickens, three chickens from each group were euthanized at 4 dpi and tissue samples (trachea, lung, pancreas, spleen, kidney and intestine) were collected for viral titration. The remaining birds were euthanized at the end of experiment. Serum samples were collected for serology tests.

### Ethics statement

All animal studies in this study were conducted in accordance to the guidelines of the Animal Care and Use Committee of Shanghai Veterinary Research Institute, and all animal studies protocols are approved by Chinese Academy of Agricultural Science (Permit number: SHVRI-Po-0120).

## Results

### Virus isolation and growth kinetics

87 out of 1000 oropharyngeal swabs collected from fowls during 2009 to 2011 were identified as influenza virus positive according to the hemagglutination assay and RT-PCR amplification of M segment. Seven viruses were determined as H3 subtype AIV based on the HI results. Further sequencing of the HA and NA gene revealed that four H3N2 and three H3N8 viruses were isolated. Consequently, the seven isolates were named as A/duck/Shanghai/120-1/2009 (H3N8, 120-1/H3N8), A/duck/Nanjing/A1591-1/2010 (H3N8, A1591-1/H3N8), A/chicken/Nanjing/B854-2/2011 (H3N8, B854-2/H3N8), A/duck/Hebei/B1645-2/2011/(H3N2, B1645-2/H3N2), A/duck/Hebei/B1646-2/2011 (H3N2, B1646-2/H3N2), A/duck/Hebei/B1647-1/2011 (H3N2, B1647-1/H3N2) and A/duck/Shanghai/74-1/2009 (H3N2, 74-1/H3N2). B854-2/H3N8 virus was isolated from chicken and all the others were from ducks. No obvious clinical signs were observed in either of the fowl. Full genomes of all seven H3 isolates were sequenced and deposited in GenBank, as accession numbers listed in Table S1.

**Figure 1:**
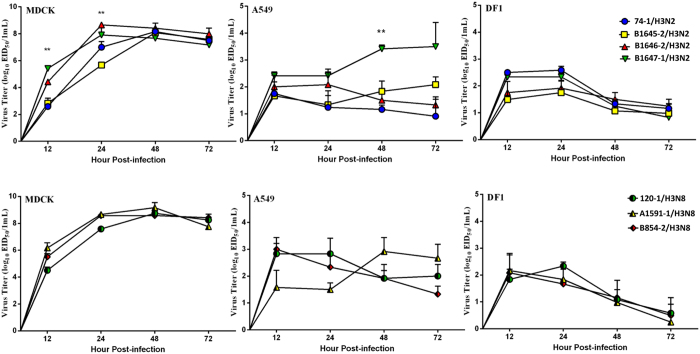
Growth properties of the H3 viruses in MDCK, A549 and DF-1 cells. The cells were infected with each virus at an MOI of 0.01. Each point on the curve indicates the mean from three independent experiments, and error bars indicates the standard errors of the mean (SEM) (*P < 0.05; **P < 0.01).

Growth kinetic results showed that all H3 viruses replicated efficiently in MDCK cells, and replicated to different titers in A549 and DF-1 cell (Fig. 1). Notably, B1647-1/H3N2 replicated more efficiently than other H3N2 viruses at 48 and 72 hours post infection in A549 cells. In DF-1 cell, all viruses replicated to lower titers compared with those in MDCK cells.

### Molecular characterization of H3 isolates

Seven H3 isolates shared the same amino acid sequence (PEKQTR/GLF) at the cleavage site between HA1 and HA2, indicating that they are low pathogenic strains. 98Y, 134G, 135K, 136S, 137G, 138A, 153W, 155V, 183H, 190E, 194L, 195Y, 224R, 225G, 226Q, 227S, 228G, and 229R were observed in hemagglutinin at the receptor-binding pocket area of all seven isolates. None of these residues have been reported to be involved in recognition of human-like receptors, suggesting that all the isolates prefer avian-like receptors. E/D at position 627/701 of the polymerase PB2 protein of the seven H3 isolates suggested that these isolates were short of efficient adaptation to mammals. According to sequences of NA, all isolates presented susceptibility to neuraminidase inhibitors (oseltamivir, zanamivir, and peramivir)^35^. Two isolates, B1645-2/H3N2 and B1646-2/H3N2 possessed an S31N mutation in the viral ion channel M2 (encoded by the M segment) that confers resistance to the M2 ion channel blockers^36^,^37^.

### Phylogenetic analysis of HA and NA genes

**Figure 2:**
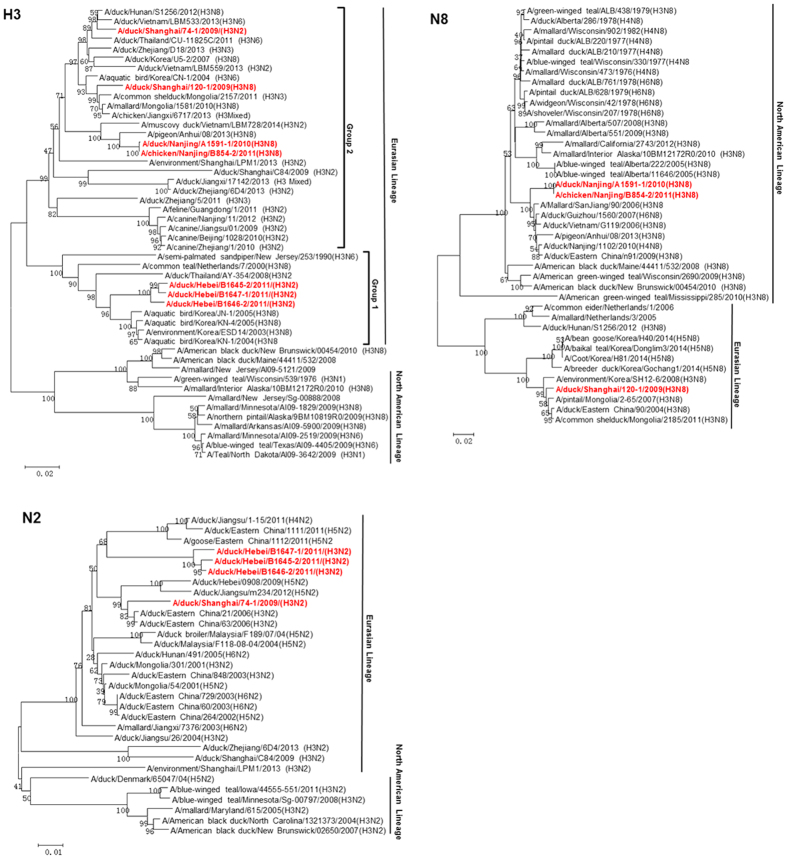
Phylogenetic trees of the H3, N2, and N8 genes of the seven H3 influenza viruses. The trees were generated by the distance based neighbor-joining method in the software MEGA 6.0. The reliability of the trees was assessed by bootstrap analysis with 1,000 replications. Horizontal distances are proportional to genetic distance. The viruses isolated in this study are in bold and red.

Based on phylogenetic trees, HA segments of seven H3 isolates are grouped into Eurasian lineage. Three H3N2 isolates from Hebei (B1645-2/H3N2, B1646-2/H3N2, B1647-1/H3N2) were clustered in group 1 including H3 subtype influenza viruses from Netherlands and Korea (Fig. 2). The remaining H3 isolates from Shanghai (120-1/H3N8, 74-1/H3N2) and Nanjing (A1591-1/H3N8, B854-2/H3N8) fell in group 2 with H3 subtype influenza viruses from Eastern China, Thailand and Vietnam. All N2 genes were clustered in Eurasian lineages, and showed close relationship with N2 gene of highly pathogenic H5N2 circulating in Eastern China (Fig. 2). Notably, N8 genes of A/duck/Nanjing/A1591-1/2010 (H3N8) and A/chicken/Nanjing/B854-2/2011 (H3N8) were grouped into the North American lineage, suggesting reassortment occurred between influenza viruses of Eurasian and North American lineages. The A/duck/Shanghai/120-1/2009 (H3N8) contains N8 gene that is close to that of highly pathogenic H5N8 virus circulating in Korea (Fig. 2).

### Phylogenetic analysis of internal genes

**Figure 3:**
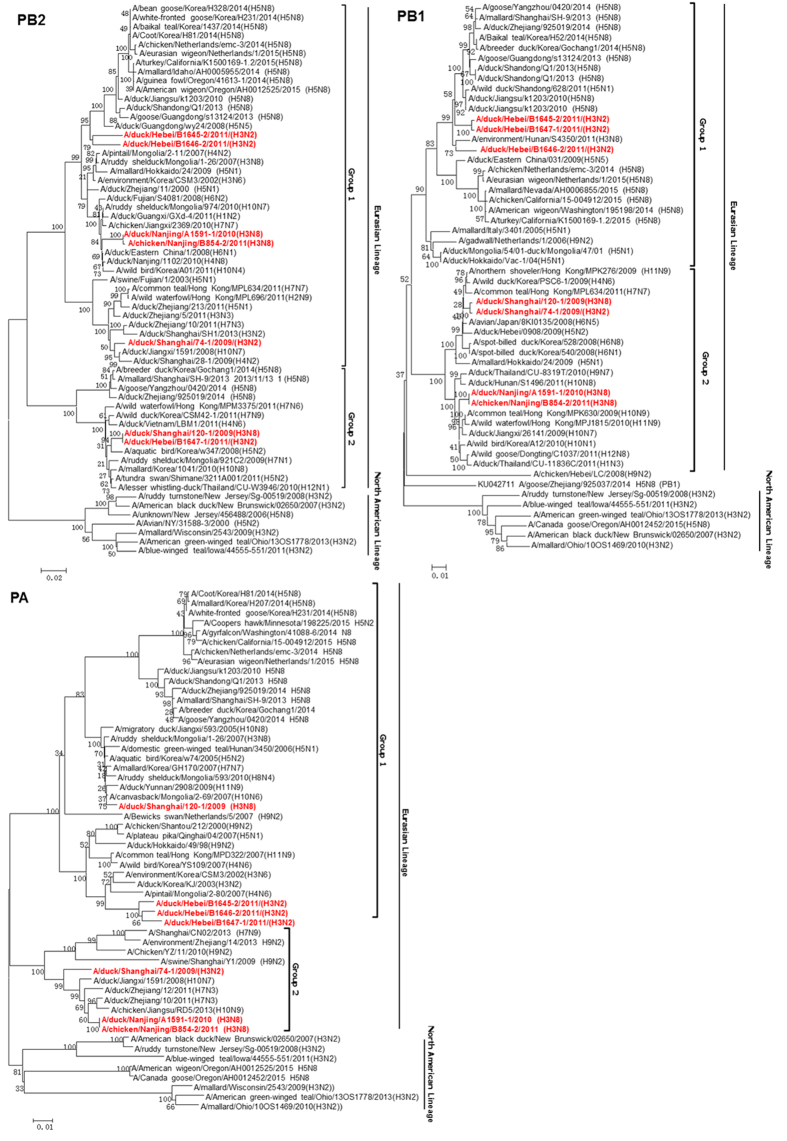
Phylogenetic trees of the PB2, PB1, and PA genes of the seven H3 influenza viruses.

**Figure 4:**
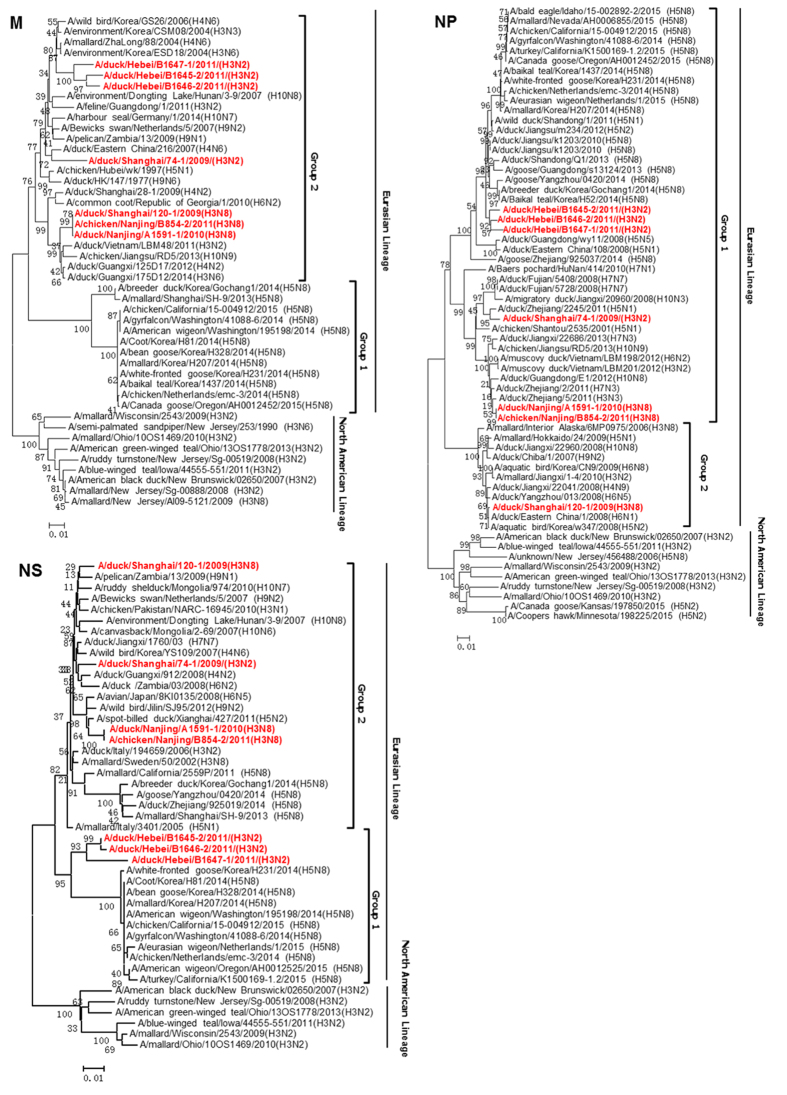
Phylogenetic trees of the NP, M, and NS genes of the seven H3 influenza viruses.

Phylogenic analysis showed that internal genes of all seven H3 isolates distributed in group 1 and group 2 within the Eurasian lineage. Notably, in group 1, the PB1, NP and NS genes of three H3N2 isolated from Hebei (B1645-2/H3N2, B1646-2/H3N2, B1647-1/H3N2) had close relationship with those of highly pathogenic H5N8 virus emerged in Korea and United States (Figs 3 and 4). Similarly, PB2 of two Hebei H3N2 (B1645-2/H3N2, B1646-2/H3N2) were grouped with H5N8 viruses (Fig. 3). The remaining genes of these H3 isolates shared high identities with influenza viruses from wild birds in group 1 and 2 (Figs 3 and 4).

PA gene of A/duck/Shanghai/120-1/2009 (H3N8) also has close relationship with the highly pathogenic H5N8 virus (Fig. 3). PA genes of 74-1/H3N2, A1591-1/H3N8, B854-2/H3N8 fell into subgroup of H9N2 and H7N9 viruses in group 2 (Fig. 3). The M genes of seven H3 isolates fell into two sub-groups in the Eurasian lineage in group 2 and have close relationship with influenza viruses from wild birds with a higher homology (Fig. 4).

### Antigenic cross-reactivity of H3 viruses

To test the antigenic cross-reactivity among H3 viruses isolated in this study, based on the phylogenetic trees of HA gene, four representative H3 isolates B1646-2/H3N2 from group 1 and 74-1/H3N2, 120-1/H3N8, and 854-2/H3N8 from group 2 were chosen for antigenic analysis. The results showed that B1646-2/H3N2 from group 1 showed low cross-reactivity with sera against viruses (74-1/H3N2, 120-1/H3N8 and 854-2/H3N8) from group 2. Similarly, antiserum against B1646-2/H3N2 had low reactivity with the viruses of 74-1/H3N2, 120-1/H3N8 and 854-2/H3N8 (Table 1). The present results indicated that two antigenically different H3 viruses are circulating in LPM in China.

### Pathogenicity in mice

**Figure 5:**
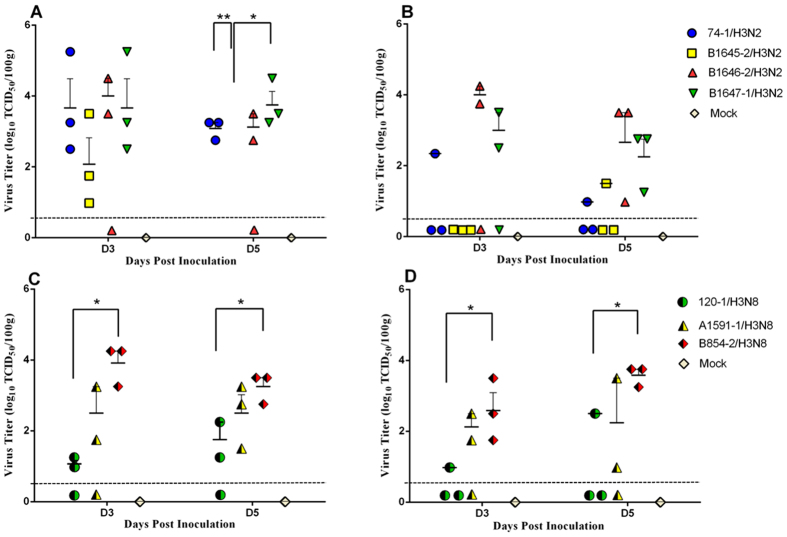
Replication of influenza virus in nasal turbinate and lung of inoculated mice. Viral titers in the lungs (**A,C**) and nasal turbinate (**B,D**) harvested at 3, and 5dpi. Viral titers were determined by EID_50_ by using embryonated chicken eggs. Error bars represent standard errors of the mean (SEM), dotted line represents the limitation of the detection. *P < 0.05, **P < 0.01.

**Figure 6:**
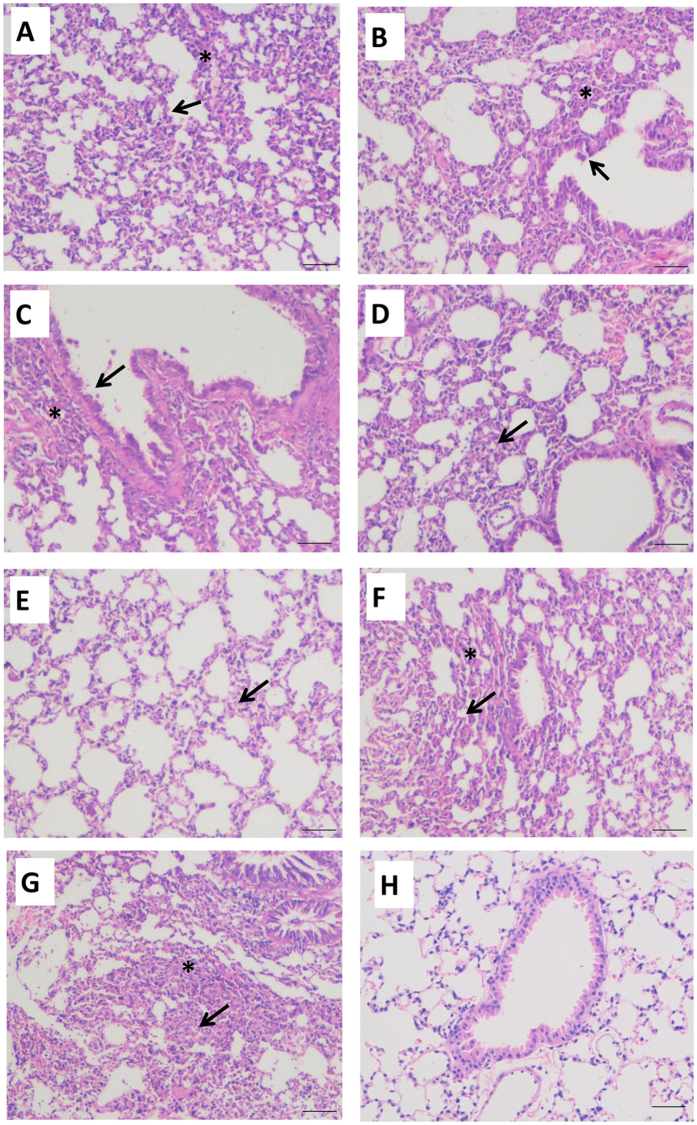
Microscopic lung sections from mice inoculated with various H3 viruses on 5 dpi. (**A**) 74-1/H3N2, damage of alveolar epithelium (arrow), lymphocytic cuffing with marked lymphocyte infiltration of the lamina propria (asterisk). (**B**) B1645-2/H3N2, mild bronchiolar epithelial damadge (arrow), mild alveolar septal thickening and light infiltration of lymphocyte (asterisk). (**C**) B1646-2/H3N2, bronchiolar epithelial attenuation (arrow), peribronchiolar lymphocytic cuffing with marked lymphocyte infiltration (asterisk). (**D**) B1647-1/H3N2, Moderate alveolar septal thickening and infiltration of lymphocyte (arrow). (**E**) 120-1/H3N8, light alveolar septal thickening (arrow). (**F**) A1591-1/H3N8, damage of alveolar epithelium (arrow), a large part of alveolar spaces were filled with lymphocyte (asterisk). (**G**) B854-2/H3N8, severe damage of alveolar epithelium (arrow), alveolar lumina and septa were filled with a number of lymphocyte (asterisk). (**H**) Mock. The scale bar indicates 50 μm in all photographs. H&E staining of the microscopic lung sections from mice necropsied at 5 dpi.

To assess the pathogenicity of the H3 isolates in mammalian host, mice were inoculated with 10^6^ EID_50_ viruses intranasally. No clinical sign was observed in either group, and all H3 isolates caused only slightly weight loss in mice (data not shown). The virus titration results showed that all the H3 isolates replicated in lung and nasal turbinate without prior adaptation (Fig. 5), while no virus was detected in other tissues (brain, spleen, and kidney). B854-2/H3N8 replicated to significantly higher titers in nasal turbinate than A1591-1/H3N8 and 120-1/H3N8 at 3 and 5 dpi (Fig. 5D). Similarly, B854-2/H3N8 also replicated more efficiently than the other two H3N8 viruses in lungs at 3 dpi (Fig. 5C). Among the four H3N2 isolates, B1646-2/H3N2 replicated to higher titers than those of B1645-2/H3N2 and 74-1/H3N2 in nasal turbinates at both 3 and 5 dpi (Fig. 5B). On the other hand, all H3 viruses replicated efficiently in mouse lungs except B1645-2/H3N2 (Fig. 5A). Based on histopathological examination, all H3 viruses caused mild bronchointerstitial pneumonia, which were characterized by infiltration of the alveolar lumen with neutrophils and by damage of the alveolar epithelium (Fig. 6). The 74-1/H3N2 infection caused damage of alveolar epithelium and marked lymphocyte infiltration in mouse lungs (Fig. 6). The 1645-2/H3N2, B1646-2/H3N2, and B1647-1/H3N2 infections resulted in mild to moderate bronchiolar epithelial damage, mild alveolar septal thickening and infiltration of lymphocyte (Fig. 6). The 120-1/H3N8 only caused light alveolar septal thickening, while A1591-1/H3N8 and B854-2/H3N8 infections caused damage of alveolar epithelium and lymphocyte infiltration (Fig. 6). Based on the microscopic lung lesion scores, mice infected by B854-2/H3N8 showed severer lung lesion than mice infected by the other two H3N8 viruses (Figure S1); mice infected by B1646-2/H3N2 virus had slighly higher lung lesion score than mice infected by the other H3N2 viruses (Figure S1).

### Pathogenicity in chickens

To evaluate the pathogenicity of H3 isolates in chickens, three representative viruses (B854-2/H3N8, 120-1/H3N8 and B1646-2/H3N2 from different groups in HA phylogenetic trees) were chosen for the chicken infection study. The results showed that no clinical sign was observed in the infected chickens, and all three viruses replicated inefficiently in chickens. B854-2/H3N8 and 120-1/H3N8 were only detected in pancreas. B1646-2/H3N2 was detected in trachea, lung, pancreas and intestine with low titers (Table S2). These three viruses shed inefficiently in chickens. Virus was detected in oropharynx swabs of one to two chickens at 3 dpi in all three groups. One chicken in each group infected with B854-2/H3N8 or B1646-2/H3N2 shed virus with relatively low titers from cloacal route at 5 dpi (Table S3). All the results suggested that duck-originated viruses have not adapted to chicken.

## Discussion

Multiple subtype influenza viruses (H3, H4, H6, H9, H10, etc.) have circulated and evolved in live poultry markets which was considered to be a prolific source of AIVs in China^38^. The H3 subtype has a wide range of host, including fowl, canine, horse, cat, canine, swine^5^,^23^,^39^,^40^,^41^,^42^,^43^,^44^. The H3 subtype can establish infection upon interspecies transmission from avian sources, posing a threat to mammals and humans. Hence, it is important to monitor the evolution of H3 AIVs in the live poultry markets. In the present study, seven H3 viruses isolated from Shanghai, Nanjing and Hebei were sequenced and analyzed. All the viruses belong to Eurasian lineage based on the HA phylogenetic trees, however the NA genes of B854-2/H3N8 and A1591/H3N8 clustered with viruses from North American lineage, which suggested reassortment event occurred between these two lineages. However, the N8 NA gene of the North American lineage was introduced into China in 1992. Studies suggested that it may be introduced by wild bird’s migration^45^,^46^.

The highly pathogenic avian influenza (HPAI) subtype H5N8 virus was introduced into Korea and North America, and Europe in 2014^47^. Studies showed that the H5N8 arose in Asia from reassortment events between HPAI subtype H5N1 virus (clade 2.3.4.4) and several low pathogenicity viruses^48^. Herby, the present results showed that PB2, PB1, NP, and NS of 1646-2/H3N2 and B1645-2/H3N2 showed close relationship with highly pathogenic H5N8 from Korea, United States and Europe. However, B1647-1/H3N2 isolated from the same location has different reassortant pattern. Its PB1, NP and NS sub-grouped with those of H5N8, but the PB2 gene was clustered with other wild bird influenza viruses. Notably, the present data indicated that the H3N2-like and H3N8-like low pathogenicity viruses might contribute internal genes to the highly pathogenic H5N8, which was reported in China in 2013^49^. Then the H5N8-like virus was introduced into South Korea during 2014, thereby caused outbreaks in wild birds and poultry farms^48^.

Phylogenetic analysis of HA genes and antigenic cross-reactivity results indicated that two antigenically different lineages H3 viruses are co-circulating in LPM in China. The H3N2 influenza viruses from group 1 had close relationship with H3 viruses circulating in Korea. The chicken study indicated that the three representative viruses (B854-2/H3N8 120-1/H3N8, and B1646-2/H3N2) from group 1 and 2 replicated and shed inefficiently in domestic chickens, despite the B854-2/H3N8 was isolated in chicken. It suggested that duck-originated viruses have not adapted chicken.

Mammalian adaptation associated residues (PB2- 627, 701) were absent in all the seven H3 viruses, although all these H3 viruses replicated efficiently in MDCK cells. Further mice study suggested that most of the isolates replicated efficiently in mice without prior adaptation. Viral titers in lungs of certain H3N2 viruses (i.e. 1646-H3N2) are comparable to those of H5N2 and H5N8 in infected mice reported recently^50^, suggesting a potential cross-species transmission of H3 influenza viruses. Notably, the three viruses of 1646-2/H3N2, B1645-2/H3N2 and B1647-1/H3N2 isolated at the same location showed different replication ability in mice. B1645-2 barely replicated in lung and nasal turbinate, which is different from the other two viruses, the underneath mechanism needs further investigation. The H3 viruses caused microscopic lung lesions in mice without prior adaptation, however all the viruses caused slight weight loss. The possible reason is that the H3 viruses were low pathogenic to mice, the inflammation induced by the infection helped to clear the virus, and it was still self-limiting and nonlethal to the mice.

Taken together, the seven H3 viruses isolated from LPM in China reassorted with different influenza viruses, indicating that intense reassortment is occurring among different subtype influenza viruses from different areas. However whether the reassortment happened in LPM or before the fowl introduced into it remain unclearly. Nevertheless co-circulation of these viruses in LPM will enhance the reassortment. Hence, continuous surveillance in LPM is warranted.

## Additional Information

**How to cite this article**: Cui, H. *et al.* Phylogenetic analysis and pathogenicity of H3 subtype avian influenza viruses isolated from live poultry markets in China. *Sci. Rep.* **6**, 27360; doi: 10.1038/srep27360 (2016).

## Supplementary Material

Supplementary Information

## Figures and Tables

**Table 1:** Antigenic analysis of H3 avian influenza viruses.

Virus	HI titers to antiserum			
	Chicken sera against 74-1/H3N2†	Chicken sera against 120-1/H3N8	Chicken sera against 854-2/H3N	Chicken sera against B1646-2/H3N2
74-1/H3N2	256	1024	1024	64
120-1/H3N8	256	1024	1024	128
854-2/H3N8	128	1024	1024	64
B1646-2/H3N2	128	128	128	512

^†^Antisera were generated from SPF chickens immunized twice with representative H3 avian influenza viruses. Homologous titers are shown in boldface.
