# Supplementary material for: Phylogenetic analysis and pathogenicity of H3 subtype avian influenza viruses isolated from live poultry markets in China
Source: Sci Rep. 2016 Jun 7;6:27360. doi: 10.1038/srep27360 (PMC4895239; doi:10.1038/srep27360)
Supplement: Supplementary Information [file srep27360-s1.doc]

# Phylogenetic analysis and pathogenicity of H3 subtype avian influenza viruses isolated from live poultry markets in China

Hongrui Cui^a^, Ying Shi^a^, Tao Ruan^a^, Xuesong Li^a^, Qiaoyang Teng^a^, Hongjun Chen^a^, Jianmei Yang^a^, Qinfang Liu^a^*, Zejun Li^a^*

^a^*Shanghai Veterinary Research Institute, Chinese Academy of Agricultural Sciences, Shanghai, People's Republic of China*

*Address for correspondence: Zejun Li, Qinfang Liu, Shanghai Veterinary Research Institute, Chinese Academy of Agricultural Sciences, 518 Ziyue Road, Shanghai 200241, P. R. China; email: [lizejun@shvri.ac.cn](mailto:lizejun@shvri.ac.cn); [liuqinfang@shvri.ac.cn](mailto:liuqinfang@shvri.ac.cn)

Supplementary Data

| Strains | Segment | | | | | | | |
| --- | --- | --- | --- | --- | --- | --- | --- | --- |
|  | PB2 | PB1 | PA | HA | NP | NA | M | NS |
| A/duck/Shanghai/120-1/2009(H3N8) | KU158937 | KU158930 | KU158923 | KU158888 | KU158909 | KU158902 | KU158895 | KU158916 |
| A/duck/Nanjing/A1591-1/2010(H3N8) | KU158938 | KU158931 | KU158924 | KU158889 | KU158910 | KU158903 | KU158896 | KU158917 |
| A/chicken/Nanjing/B854-2/2011(H3N8) | KU158939 | KU158932 | KU158925 | KU158890 | KU158911 | KU158904 | KU158897 | KU158918 |
| A/duck/Hebei/B1645-2/2011/(H3N2) | KU158940 | KU158933 | KU158926 | KU158891 | KU158912 | KU158905 | KU158898 | KU158919 |
| A/duck/Hebei/B1646-2/2011/(H3N2) | KU158941 | KU158934 | KU158927 | KU158892 | KU158913 | KU158906 | KU158899 | KU158920 |
| A/duck/Hebei/B1647-1/2011/(H3N2) | KU158942 | KU158935 | KU158928 | KU158893 | KU158914 | KU158907 | KU158900 | KU158921 |
| A/duck/Shanghai/74-1/2009/(H3N2) | KU158943 | KU158936 | KU158929 | KU158894 | KU158915 | KU158908 | KU158901 | KU158922 |

**Table S1. Accession numbers of nucleotide sequences for all H3 influenza viruses in GeneBank**

| Virus | Virus Titters (log_10_ EID_50_ /1mL) | | | | | |
| --- | --- | --- | --- | --- | --- | --- |
|  | Trachea | Lung | Pancreas | Intestine | Kidney | Spleen |
| 120-1/H3N8 | 0/3 (NT) | 0/3 (N.A.) | 1/3 (0.75) ^a^ | 0/3 (N.A.) | 0/3 (N.A.) | 0/3 (N.A.) |
| B854-2/H3N8 | 0/3 (NT) | 0/3 (N.A.) | 1/3 (1.25) | 0/3 (N.A.) | 0/3 (N.A.) | 0/3 (N.A.) |
| B1646-2/H3N2 | 1/3 (2.5) | 1/3 (1.5) | 2/3(1.00±0.25) | 2/3(1.13±0.37) | 0/3 (N.A.) | 0/3 (N.A.) |

**Table S2. Virus titers in tissues of infected chickens.** Chickens (12-week-old) were inoculated intranasally with the viruses at a dose of 10^6^ EID_50_. Three from each group were euthanized at 4 dpi, and tissue samples (trachea, lung, pancreas, intestine, kidney and spleen) were taken aseptically for virus titration. a: One out of three infected ducks are positive for virus detection, the titer values were shown as (mean± SEM); N.A.: virus could not be detected in all three chickens.

| Virus | Virus Titters (log_10_ EID_50_ /1mL) | | | | | | |  |
| --- | --- | --- | --- | --- | --- | --- | --- | --- |
|  | D3 | |  | D5 | |  | D7 | |
|  | oropharynx swab | cloacal swab |  | oropharynx swab | cloacal swab |  | oropharynx swab | cloacal swab |
| 120-1/H3N8 | 2/4(1.50±0.00)^a^ | 0/4 ( N.A.) |  | 1/4(1.5) | 0/4 ( N.A. ) |  | 0/4 (N.A.) | 0/4 (N.A.) |
| B854-2/H3N8 | 1/4 (0.25) | 0/4 ( N.A. ) |  | 0/4 ( N.A. ) | 1/4 (0.25) |  | 0/4(N.A.) | 0/4 (N.A.) |
| B1646-2/H3N2 | 2/4(0.88±0.63) | 1/4 (0.25) |  | 0/4 ( N.A. ) | 1/4(0.75) |  | 2/4(0.1.63±0.13) | 1/4 (1.5) |

**Table S3. Virus titers in oropharynx swabs and cloacal swabs of infected chickens.** Chickens (12-week-old) were inoculated intranasally with the viruses at a dose of 10^6^ EID50. Oropharynx swabs and cloacal swabs were collected at 3, 5 and 7 dpi for virus titration. a: Two out of four infected chickens are positive for virus detection, the titer values were shown as (mean± SEM); N.A.: virus could not be detected in all four chickens.


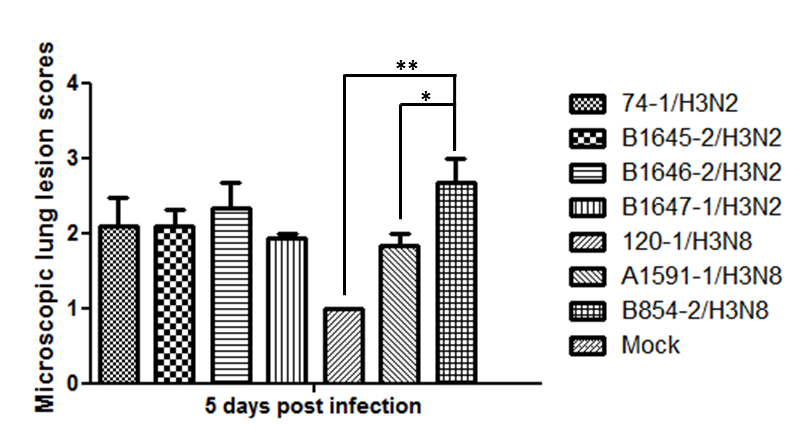


**Figure S1. Microscopic lung lesion scores of mice inoculated with H3 influenza viruses.** All data represent the mean ± SEM of microscopic lung lesion scores (*: P < 0.05, **: P < 0.01).
